# Supplementary material for: Maternal Vaginal Pathogenic Bacterial Colonization and Early-Onset Neonatal Sepsis in Mwanza City, Tanzania: A Prospective Cohort Study
Source: medRxiv. 2026 Oct 26:2026.10.22.26366266. Preprint. [Version 1] doi: 10.64898/2026.10.22.26366266 (PMC13734031; doi:10.64898/2026.10.22.26366266)
Supplement: Supplementary file 1 [file NIHPP2026.10.22.26366266v1-supplement-1.pdf]

S1 Table: Demographic characteristics of the pregnant women stratified by neonatal sepsis (N=580)

Variables	N (%)	Neonatal sepsis		
		No (N=491) n (%)	Yes (N=89) n (%)	χ^2 /t-test (p-value)
**Maternal age (median, IQR)**	29.5, 25-34	29, 25-34	30, 27-35	0.13
**Age (in years)**				0.84
17-24	123(21.2)	106(86.2)	17(13.8)	
25-35	361(62.2)	305(84.5)	56(15.5)	
36-44	96(16.6)	80(83.3)	16(16.7)	
**Marital status**				0.42
Not married	94(16.2)	77(81.2)	17(18.1)	
Married	486(83.8)	414(85.2)	72(14.8)	
**Education level**				0.18
Primary education	226(39.0)	184(81.4)	42(18.6)	
Secondary education	218(37.6)	187(85.8)	31(14.2)	
College and above	136(23.4)	120(88.2)	16(11.8)	
**Occupation**				0.66
Not employed	160(27.6)	133(83.1)	27(16.9)	
Employed	95(16.4)	83(87.4)	12(12.6)	
Entrepreneur	325(56.0)	275(84.6)	50(15.4)	
**Practice douching**				0.61
No	314(54.1)	268(85.4)	46(14.7)	
Yes	266(45.9)	223(83.8)	43(16.2)	
**Body mass index (mean, SD)**	25.02, 1.3	25.2(11.0)	24.2(5.2)	0.39
BMI				0.38
Normal	309(53.3)	264(85.4)	45(14.6)	
Underweight	35(6.0)	26(74.3)	9(25.7)	

Overweight	153(26.4)	130(85.0)	23(15.0)	
Obesity	83(14.3)	71(85.5)	12(14.5)	
**Weight gain (mean, SD)**	7.3, 4.9	7.3(4.9)	7.0(4.0)	0.51
**Weight gain**				0.28
Too little (<8kg)	359(61.9)	306(85.2)	53(14.8)	
Normal (8kg – 12 kg)	155(26.7)	126(81.3)	29(18.7)	
Too much (>12kg)	66(11.4)	59(89.4)	7(10.6)	
**Frequency of sex per week (mean, SD)**	2.4, 1.9	2.4(1.9)	2.3(1.2)	0.56
**Sex frequency per week**				0.33
None	28(4.8)	27(96.4)	1(3.6)	
Once	135(23.3)	110(81.5)	25(18.5)	
2 times	170(29.3)	145(85.3)	25(14.7)	
3 times	143(24.7)	119(83.2)	24(16.8)	
4 times and above	104(17.9)	90(86.5)	14(13.5)	
**Healthcare facility**				0.27
Bugando Medical Centre	337(58.1)	289(85.8)	48(14.2)	
Buzuruga Healthcare Centre	46(7.9)	40(87.0)	6(13.0)	
Igoma Healthcare Centre	48(8.3)	41(85.4)	7(14.6)	
Nyamagana District Hospital	54(9.3)	40(74.1)	14(25.9)	
Sekou Toure Regional Hospital	95(16.4)	81(85.3)	14(14.7)	
**Area of residence**				**0.005**
Outside Mwanza City	72(12.4)	53(73.6)	19(26.4)	
Within Mwanza City	508(87.6)	438(86.2)	70(13.8)	

S2 Table: Clinical characteristics of the pregnant women stratified by neonatal sepsis (N=580)

**Variables**	**n (%) or mean (SD**	**Neonatal sepsis**		
		**No (N=491) n (%)**	**Yes (N=89) n (%)**	**χ^2 /t-test (p-value)**

**Gravidity (Mean, SD)**	3.2, 1.8	3.1(1.8)	3.2(1.6)	0.69
**Gravidity**				0.37
Primigravida	125(21.6)	109(87.2)	16(12.8)	
Multigravida	455(78.4)	382(84.0)	73(16.0)	
**Parity (Mean, SD)**	1.8, 1.6	1.8(1.6)	2.0(1.5)	0.45
**Parity**				0.38
Nulliparous	156(26.9)	136(87.2)	20(12.8)	
Primiparous	111(19.1)	96(86.5)	15(13.5)	
Multiparous	313(54.0)	259(82.8)	54(17.3)	
**Number of antenatal care (ANC) (Mean, SD)**	4.9, 1.7	4.9(1.7)	4.8(1.9)	0.81
**ANC visits**				0.88
Below 8 visits	543(93.6)	460(84.7)	83(15.3)	
8 visits and above	37(6.4)	31(83.8)	6(16.2)	
**Premature Rupture of Membranes (PROM)**				0.17
No	530(91.4)	452(85.3)	78(14.7)	
Yes	50(8.6)	39(78.0)	11(22.0)	
**Other clinical findings (Yes)**				
Anaemia	205(35.3)	168(82.0)	37(18.0)	0.18
History of diabetes mellitus	57(9.8)	46(80.7)	11(19.3)	0.38
Gestational diabetes mellitus (n=523)	9(1.7)	9(100.0)	0(0.0)	0.21
Malaria	31(5.3)	20(64.5)	11(35.5)	**0.01**
Urinary Tract Infection (UTI)	129(22.2)	104(80.6)	25(19.4)	0.15
Preeclampsia	52(9.0)	40(76.9)	12(23.1)	0.11
Schistosomiasis	20(3.5)	18(90.0)	2(10.0)	0.5
History of antibiotic use	136(23.5)	115(84.6)	21(15.4)	0.97
Previous history of genital infection	263(45.3)	221(84.0)	42(16.0)	0.70
History of previous neonatal death	31(5.3)	27(87.1)	4(12.9)	0.69
History of previous early neonatal sepsis	29(5.0)	25(86.2)	4(13.8)	0.81
**HIV status**				**<0.01**
Negative	382(65.9)	339(88.7)	43(11.3)	
Positive	198(34.1)	152(76.8)	46(23.2)	
**Duration on ART (mean years, SD) (n=198)**	4.5, 4.3	4.7(4.6)	3.7(3.0)	0.19
**ART Duration (n=198)**				0.59
Below 1 year	46(23.2)	35(76.1)	11(23.9)	

1-5 years	84(42.4)	62(73.8)	22(26.2)	
**6 years and above**	68(34.3)	55(80.9)	13(19.1)	
**CD4 count (mean, SD) (n=198)**	618.6, 1.8	623.1(148.7)	603.7(167.5)	0.45
Less than 500 counts	52(26.3)	44(84.6)	8(15.4)	0.12
500 counts and above	146(73.7)	108(74.0)	38(26.0)	
**Vaginal colonization status**				**<0.01**
Not colonized	172(29.7)	168(97.7)	4(2.3)	
Colonized	408(70.3)	323(79.2)	85(20.8)	
**Number of pathogenic organisms (n=408)**				**0.03**
Single	323(79.2)	263(81.4)	60(18.6)	
Double	85(20.8)	60(70.6)	25(29.4)	
**Phenotypic resistance (n=114/408)**				0.70
MRSA	68(59.7)	49(72.1)	19(27.9)	
MRSA + iMLSB	32(28.1)	25(78.1)	7(21.9)	
ESBL	12(10.5)	8(66.7)	4(33.3)	
iMLSB	2(1.8)	2(100.0)	0(0.0)	
**Maternal sepsis**				**<0.01**
No	537(92.6)	463(86.2)	74(13.8)	
Yes	43(7.4)	28(65.1)	15(34.9)	
**Maternal antibiotic use before delivery**				**<0.01**
No	328(56.6)	289(88.1)	39(11.9)	
Yes	252(43.5)	202(80.2)	50(19.8)	
**Phenotypic concordance**				**<0.01**
No	2(8.3)		2(8.3)	
Yes	22(91.7)		22(91.7)	

S3 Table: Intrapartum & Neonatal characteristics stratified by neonatal sepsis (N=580)

Variables	n (%) or mean	Neonatal sepsis status

	(SD)	No n (%)	Yes n (%)	χ^2 /t-test (p-value)
**Duration of labour in hours (Mean, SD)**	8.6(4.3)	8.6(4.3)	8.0(4.7)	0.18
**Duration of rupture of membrane in hours (Mean, SD)**	6.0(10.2)	5.9(9.4)	6.9(13.9)	0.37
**Number of vaginal Examinations (Mean, SD)**	2.8(1.5)	2.8(1.5)	2.7(1.5)	0.27
**Mode of delivery**				0.11
Spontaneous vaginal delivery	400(69.0)	345(86.2)	55(13.8)	
Caesarean section	180(31.0)	146(81.1)	34(18.9)	
**Type of Caesarean section (n=180)**				0.73
Elective	59(32.8)	47(79.7)	12(20.3)	
Emergency	121(67.2)	99(81.8)	22(18.2)	
**Sepsis status (n=89)** Clinical-diagnosed	65(73.0)		65(73.0)	
Culture-confirmed	24(27.0)		24(27.0)	
**Bacterial isolates (n=24)**				
E. coli	14(58.3)		14(58.3)	
K. pneumoniae	5(20.8)		5(20.8)	
S. aureus	5(20.8)		5(20.7)	
**Birth-weight (Mean, SD)**	3.0(0.5)	3.0(0.5)	2.9(0.7)	**0.01**
**Birth-weight**				**0.01**
Normal birthweight (>=2.5kg)	505(87.1)	435(86.1)	70(13.9)	
LBW (<2.5kg)	75(12.9)	56(74.7)	19(25.3)	
**LBW (n=75)**				**0.01**
Low (1.5kg-2.4kg)	67(89.4)	53(79.1)	14(20.9)	
Very Low (<1.4kg)	8(10.6)	3(37.5)	5(62.5)	
**Gestational age at birth (Mean, SD)**	38.4(2.3)	38.4(2.3)	37.9(2.6)	0.08
**Gestational age at birth**				0.27
Post-term (Above 42 weeks)	19(3.3)	17(89.5)	2(10.5)	
Term (37-42 weeks)	475(81.9)	406(85.5)	69(14.5)	

Premature (<37 weeks)	86(14.8)	69(79.1)	18(20.9)	
**Premature birth (n=86)**				0.59
Premature late term (34-36 weeks)	64(74.4)	52(81.3)	12(18.8)	
Premature moderate term (32-33 weeks)	9(10.5)	6(66.7)	3(33.3)	
Premature very preterm (28-32 weeks)	13(15.1)	10(76.9)	3(23.1)	

S4 Table: Incidence of Neonatal Sepsis by Clinical and Maternal Characteristics

Variable	Total	Neonatal sepsis	Person days	Rates (per 100 person-days)	95% CI
**Colonization status**					
Not colonized	172	4	1184	0.34	0.16-0.90
Colonized	408	85	2494	3.41	2.76-4.22
**Number of bacteria (n=408)**					
Single	323	18	2013	0.89	0.56-1.42
Double	85	6	481	1.25	0.56-2.78
**Type of bacteria (n=408)**					
Gram negative	209	17	1287	1.32	0.82-2.12
Gram positive	199	7	1207	0.58	0.28-1.22
**Phenotypic resistance (n=114/408)**					
MRSA	68	5	398	1.26	052-3.01
MRSA + iMLSB	32	2	193	1.04	0.26-4.14
ESBL	12	3	69	4.34	1.40-13.48
iMLSB	2	-	-	-	-
**Maternal sepsis**					
No	537	74	3431	2.16	1.72-2.71
Yes	43	15	247	6.07	3.66-10.07
**Maternal antibiotic use before delivery**					
No	328	39	2133	1.83	1.34-2.50
Yes	252	50	1545	3.24	2.45-4.27
**HIV status**					
Negative	382	43	2485	1.73	1.28-2.33
Positive	198	46	1193	3.86	2.89-5.15
**TOTAL**	580	89	3678	**2.42**	**1.97-2.98**

Table 5: Proportional Hazard Assumption

Variables	rho	chi	p-value
Wash underpants with disinfectant	-0.13201	1.57	0.21
Malaria	0.01463	0.02	0.88
Duration of labour	0.01286	0.02	0.90
Birth-weight	-0.07025	0.45	0.50
HIV status	0.08572	0.71	0.39
Colonization status	0.10730	1.04	0.31
Maternal sepsis	0.22181	**4.43**	**0.04**
**Overall model**		**9.08**	**0.24**

Abbreviations: chi=Chi-square value; Rho=residual hazard coefficient; p-value=probability value

Table 6: Maternal and Neonatal factors associated with neonatal sepsis

Variables	Unadjusted HR		Adjusted HR	
	HR, 95% CI	p-value	HR, 95% CI	p-value
**Age (in years)**				
17-24	1			
25-35	1.11, 0.65-1.92	0.69		
36-44	1.21, 0.61-2.39	0.59		
**Wash underpants with disinfectant**				
No	1		1	
Yes	1.69, 0.88-3.27	0.12	1.57, 0.81-3.06	0.18
**PROM**				
No	1			
Yes	1.49, 0.79-2.81	0.21		
**Anaemia**				
No	1			
Yes	1.30, 0.86-1.99	0.22		
**Malaria**				
No	1		1	
Yes	2.78, 1.48-5.23	0.001	**2.74, 1.43-5.22**	**0.002**
**UTIs**				
No	1			
Yes	1.43, 0.90-2.26	0.13		

**Preeclampsia**				
No	1			
Yes	1.60, 0.87-2.94	0.13		
**Duration of labour**	0.97, 0.92-1.02	0.18	0.96, 0.92-1.10	0.07
**Mode of delivery**				
Spontaneous vaginal delivery	1			
Caesarean section	1.42, 0.93-2.18	0.12		
**Birth-weight**				
Normal birthweight (>=2.5kg)	1		1	
Low birthweight (<2.5kg)	1.93, 1.17-3.21	0.001	1.54, 0.91-2.62	0.11
**Gestation age at birth**				
Post-term (Above 42 weeks)	0.72, 0.18-2.94	0.65		
Term (37-42 weeks)	1			
Premature (<37 weeks)	1.48, 0.88-2.49	0.14		
**HIV status**				
Negative	1		1	
Positive	2.15, 1.42-3.27	<0.001	**1.76, 1.14-2.73**	**0.01**
**Colonization status**				
Not colonized	1		1	
Colonized	9.63, 3.53-26.25	<0.001	**9.52, 3.47-26.09**	**<0.001**
**Maternal antibiotic use before delivery**				
No	1			
Yes	1.73, 1.14-2.63	0.010		
